# Burden and Characteristics of RSV-Associated Hospitalizations in Switzerland: A Nationwide Analysis from 2017 to 2023

**DOI:** 10.3390/v17111407

**Published:** 2025-10-23

**Authors:** Elisa D. Bally-von Passavant, Neetha Joseph, Nike Julia Kräutler, Daphne McCarthy-Pontier, Giorgia Lüthi-Corridori, Fabienne Jaun, Jörg D. Leuppi, Maria Boesing

**Affiliations:** 1 University Institute of Internal Medicine, Kantonsspital Baselland, CH-4410 Liestal, Switzerland; 2 Faculty of Medicine, University of Basel, CH-4056 Basel, Switzerland; 3 Moderna Switzerland GmbH, CH-4052 Basel, Switzerland

**Keywords:** respiratory syncytial virus (RSV), hospitalization, length of hospital stay, mortality, intensive care, mechanical ventilation, vaccination, comorbidities

## Abstract

Respiratory syncytial virus (RSV) is a major cause of respiratory illness, particularly in children, yet its burden in adults—especially in older adults—remains under-recognized. We analyzed RSV-related hospitalizations in Switzerland from 2017 to 2023 using national data from the Federal Statistical Office, including cases with RSV coded as either a primary or secondary diagnosis. Over 35,000 RSV-related hospitalizations were recorded. The highest incidence occurred in children under 10 years (390 per 100,000/year), with a second peak in adults ≥ 80 years (151 per 100,000/year). Older adults (≥60 years) accounted for more than 9700 hospitalizations overall, with an average of over 16,600 total hospital days per year. Average length of stay (LOS) was shortest in young children (4.6 days) and highest in adolescents (13.9 days), while in adults, it increased from 6.8 days (age 20–29) to 12.3 (age ≥ 80). Mechanical ventilation rates peaked at 12.6% in 60–69 year olds, and in-hospital mortality at 7.1% in those ≥80 years. In adults, RSV was more often recorded as a secondary diagnosis and commonly associated with chronic comorbidities, including chronic obstructive pulmonary disease, heart failure, kidney disease, and diabetes. Frailty-related diagnoses—such as cognitive or motor impairment, delirium, and need for nursing care—were also frequent. These findings highlight the importance of improved adult RSV surveillance and targeted prevention strategies in high-risk populations.

## 1. Introduction

Respiratory syncytial virus (RSV) is an enveloped RNA virus that primarily infects the respiratory tract. It can cause symptoms ranging from mild colds to more severe lower respiratory tract infections such as bronchiolitis and pneumonia, with severe cases particularly affecting infants, young children, and older adults [1]. In children, particularly in infants, RSV infection typically begins with upper respiratory symptoms such as rhinorrhea, cough, and fever, but may progressively evolve into more severe manifestations including tachypnea, dyspnea, and feeding difficulties [2]. Among RSV-hospitalized infants, approximately 60–85% develop bronchiolitis or pneumonia, and 1–10% require admission to intensive care units, while in-hospital mortality remains below 1% in high-income countries [3,4,5]. In adults aged 18–64 years, RSV infection generally manifests as a mild upper respiratory illness characterized by cough, sore throat, rhinorrhea, and headache [6,7]. Severe complications are uncommon in this age group, however, in individuals with underlying cardiopulmonary comorbidities, pneumonia occurs in approximately 10–15% of cases, and exacerbations of chronic cardiac or pulmonary disease are reported in up to 20% of RSV-positive hospitalizations [7]. In older adults (≥65 years), the frequency and severity of complications increase substantially: Pneumonia develops in approximately 30–40% of hospitalized RSV-positive patients, respiratory failure or the need for intensive care in 20–25%, and case-fatality proportions range between 8–10% [7,8].

The World Health Organization (WHO) estimates that RSV causes 33 million lower respiratory tract infections, 3 million hospitalizations, and approximately 59,600 deaths annually among children aged ≤5 years [9]. Despite its clinical importance, RSV surveillance and reporting remain inconsistent across Europe, with several countries—including Switzerland—yet to designate RSV as a notifiable disease [10].

In Switzerland, RSV surveillance exists but has traditionally focused on young children, who are known to experience high infection rates and hospitalizations [1,11]. However, this pediatric-centric approach may overlook a substantial burden of disease in adults, particularly older adults [12,13,14]. Systematic reviews suggest that RSV hospitalizations in adults may be underreported by factors ranging from 1.5 to over 20 [12,13,14]. Evidence suggests that there is a significant underestimation of adult RSV burden due to factors such as low awareness and testing rates, reduced assay sensitivity in adults, and lack of standardized case definitions [12,13,14,15,16,17,18]. Adult patients often present later in the disease course, when the virus is already in the lower airways, making standard upper respiratory swabs more likely to yield false negative results [12,16,17]. Additionally, lower viral loads and shorter viral shedding in adults complicate detection [19]. While several laboratory methods can confirm RSV infection, nucleic acid amplification tests, particularly real-time reverse transcription PCR, are highly sensitive and the most widely used [16,20]. In Switzerland, RSV diagnosis is primarily based on PCR-based methods, while rapid antigen tests are available but used less frequently in adults due to their lower sensitivity [11,20].

A growing body of literature highlights the clinical relevance of RSV in adult and immunocompromised populations. RSV is increasingly recognized as an important cause of medically attended acute respiratory illness in multimorbid patients [21]. Comorbidities such as hematologic malignancies, chronic heart failure, COPD, and other chronic lung or cardiovascular conditions can exacerbate RSV disease and increase the risk of severe outcomes [22,23,24,25,26]. Immunocompromised individuals, including those with cancer or transplant recipients, appear to be particularly vulnerable, with elevated hospitalization and mortality rates. These findings are especially relevant in older adults, among whom chronic comorbidities such as obesity, cardiovascular disease, and chronic lung disease are particularly common [27].

A recent Swiss study by Stucki et al. [28] used national hospital data from 2003 to 2021 to evaluate RSV-associated hospitalizations, focusing on RSV infection as a primary diagnosis, mainly in children. While confirming the expected high RSV burden in infants, the study likely underestimates the true adult burden, as it excluded cases with RSV infection as a secondary diagnosis, which is frequently the case in older and multimorbid patients [28].

To address these gaps, our study aimed to assess RSV-related hospitalizations across all age groups in Switzerland using data from the Swiss Federal Statistical Office (FSO) for the period 2017–2023. This more recent timeframe reflects increased awareness and improved RSV testing, particularly in adults. By including cases with RSV infection coded as either primary or secondary diagnosis, our analysis provides a more comprehensive picture of RSV-associated morbidity. Special emphasis is placed on older adults, in whom RSV infection is often overlooked despite a disproportionate burden of severe outcomes.

## 2. Materials and Methods

### 2.1. Data Collection and Analysis

This study used hospitalization data aggregated by age groups from the Swiss FSO. Specifically, all hospitalizations between 2017 and 2023 with a primary or secondary diagnosis of RSV infection, identified by the following four ICD-10 diagnosis codes, were included in the analyses: B97.4 (RSV as cause of diseases classified in other chapters), J12.1 (pneumonia caused by RSV), J20.5 (acute bronchitis caused by RSV), and J21.0 (acute bronchiolitis due to RSV). These codes represent all specific ICD-10 codes for RSV-associated hospitalizations in Swiss hospital data. Age groups were defined in bins of 10 years (0–9, 10–19, 20–29, 30–39, 40–49, 50–59, 60–69, 70–79, and 80+ years). For each age group, hospitalizations with a RSV-related primary or secondary diagnosis were analyzed for annual numbers of hospitalizations, length of hospital stay (LOS), intensive care unit (ICU) admissions, mechanical ventilations, and in-hospital mortality. Analyses were conducted using both absolute and relative frequencies, as well as rates per 100,000 inhabitants.

Data on population demographics was retrieved by year and calculated by age group based on data published by the FSO per age year [29].

### 2.2. Statistical Methods

Absolute numbers and mean LOS per age group were provided by the FSO. Swiss population numbers per age group as of 31st of December of each year were retrieved from data published by the FSO [29]. We conducted descriptive analyses such as the determination of relative frequencies and averages per age group. To account for differences in population size across age groups, hospitalization incidence rates were additionally expressed per 100,000 inhabitants per age group. This approach allows for direct comparison across age strata and better reflects the relative disease burden in the population. To better capture the overall healthcare burden of RSV-related hospitalizations, the total number of hospitalization days per year and age group were calculated by multiplying the number of hospitalizations by the average LOS. All calculations and figures were created with Excel 2016. For data protection reasons, the FSO did not provide values for case numbers between 1 and 3. In these cases, we used the lowest possible value of 1 in the calculations.

### 2.3. Ethical Considerations

This study was conducted in accordance with the ethical standards of the Declaration of Helsinki. Ethical consent was not required, as the data were anonymized and collected as part of routine healthcare operations.

## 3. Results

### 3.1. Epidemiology

Between 2017 and 2023, a total of 35,489 RSV-related hospitalizations were documented in Switzerland, corresponding to an average of approximately 5100 hospitalizations per year. While data on the implemented diagnostic procedures were not routinely reported to the FSO, in Switzerland, RSV is mostly diagnosed using PCR testing. Supplementary Figure S1 presents the annual absolute numbers, highlighting age-specific differences across years. A clear year-to-year variation was observed, most notably during the COVID-19 pandemic. In 2020, hospitalization numbers dropped well below average to 3025 cases. A marked rebound followed in 2021, driven primarily by a pronounced wave in children aged 0–9 years, with 4505 hospitalizations compared to 2700–3500 in the pre-pandemic years (2017–2019). In contrast, RSV-related hospitalizations among older adults did not return to pre-pandemic levels until 2022. Among individuals aged ≥80 years, case numbers rose from <100 in 2021 to approximately 1100 in 2022 and 2023, exceeding the pre-pandemic range of 600–900. In addition to pandemic-associated fluctuations, an overall increasing trend in RSV-related hospitalizations was observed across the study period (Supplementary Figure S1). This likely reflects greater clinical awareness and more widespread diagnostic testing in adult populations, particularly among older individuals with comorbidities.

**Figure 1:**
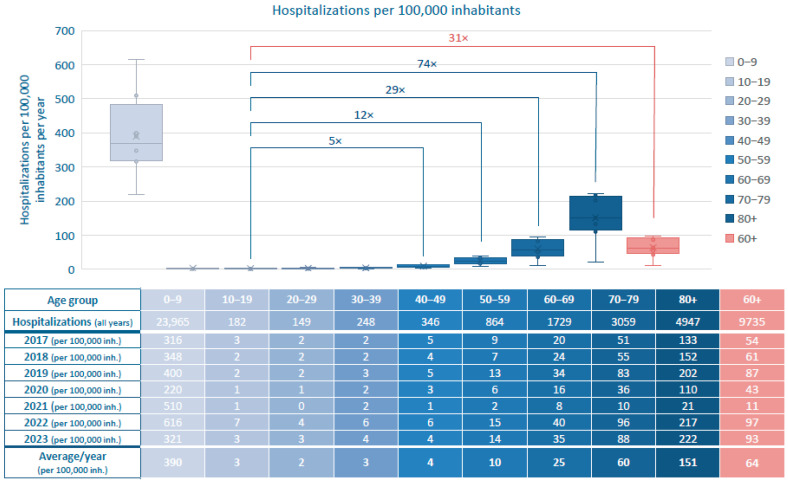
Annual and average incidence of RSV-related hospitalizations per 100,000 inhabitants, stratified by age group in 10-year increments in Switzerland, 2017–2023. All ages, including children, adolescents and adults. Fold-change in hospitalization rates per adult age group compared to the 20–29-year reference group.

Figure 1 summarizes the incidence of RSV-related hospitalizations in 2017–2023, stratified by age group in 10-year increments and expressed per 100,000 inhabitants.

The highest hospitalization incidence by far was observed in children aged 0–9 years, with an annual mean of 390 cases per 100,000 population. Hospitalization incidence declined sharply in older children and young adults (aged 10–49 years), with ≤4 cases per 100,000 annually. From age 50 onward, incidence increased steadily with rising age from 10 (ages 50–59) to 25 (ages 60–69) and 60 (ages 70–79), peaking at 151 in individuals aged ≥80 years per 100,000 population.

Comparison among adults only shows a marked increase of hospitalizations from the age of 50 onward with rates 5- to 74-fold higher than in young adults (20–29 years) and in all 60+ it was increased by 31-fold. These findings highlight the substantial and age-dependent rise in RSV-related hospitalizations across the adult population.

### 3.2. Outcome

#### 3.2.1. Length of Hospital Stay of RSV-Related Hospitalizations 2017–2023

**Figure 2:**
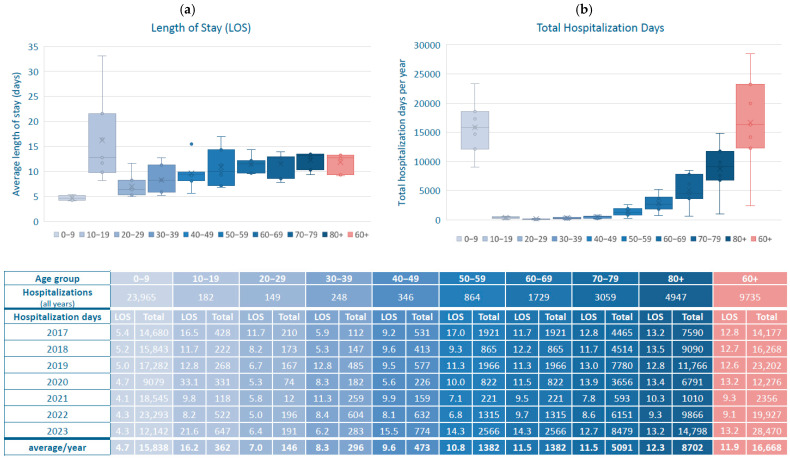
Length of hospital stay (LOS) for RSV-related hospitalizations, stratified by age group in 10-year increments in Switzerland, 2017–2023. (**a**) Average mean LOS in days is shown for each age group; (**b**) Total hospitalization days (multiplication of LOS with number of hospitalizations) for RSV-related hospitalizations per age group and year, as well as the average over the years 2017–2023. Statistical significance assessed by *t*-test. RSV: respiratory syncytial virus, LOS: length of hospital stay.

Figure 2a presents the average LOS for RSV-related hospitalizations in Switzerland by age group from 2017 to 2023. Children aged 0–9 years had the shortest hospital stays (4.6 days), while LOS increased with age in adults: 6.8 days (20–29 years), 8.4 days (30–39 years), 9.6 days (40–49 years), 11.2 days (50–59 years), 11.6 days (60–69 years and 70–79 years) and 12.3 days (≥80 years), representing a 2.4- to 2.7-fold increase compared to young children.

Figure 2b illustrates the total hospitalization days per age group. Children aged 0–9 years contributed the highest absolute number with an average of 15,838 days per year. However, older adults (aged ≥60 years) collectively accounted for an even larger burden, averaging 16,668 days per year, with those aged ≥80 years alone exceeding 8700 days annually.

#### 3.2.2. RSV-Related Hospitalizations Admitted to Intensive Care Units 2017–2023

**Figure 3:**
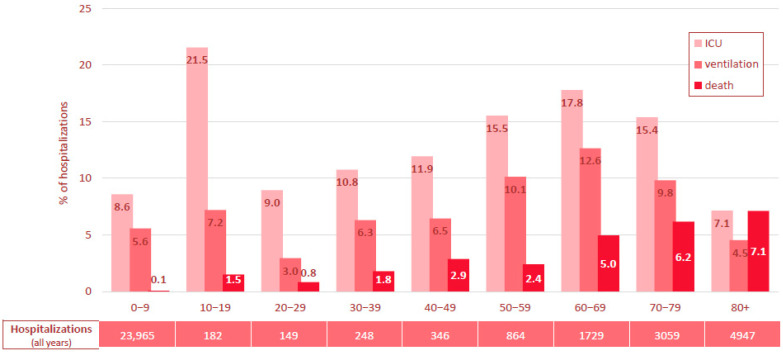
Average rate of ICU admission, mechanical ventilation, and in-hospital mortality in % of overall RSV-related hospitalizations in Switzerland, 2017–2023, stratified by age group in 10-year increments. ICU: intensive care unit admission, Hospitaliz.: Hospitalizations, RSV: respiratory syncytial virus.

Between 2017 and 2023, a total of 3441 ICU admissions were recorded in RSV-related hospitalizations in Switzerland, showing a distinct age-related pattern (Supplementary Figure S2). Children aged 0–9 years accounted for the largest absolute number of ICU admissions with 166 to 473 admissions annually though their relative ICU admission rate was comparatively low (8.6% Figure 3, Supplementary Figure S3).

Adolescents (10–19 years) had the highest ICU admission rate (21.5%), despite a low number of RSV-related hospitalizations (10–64 cases per year). In adults, ICU admission rates increased with age from 9.0% (20–29 years) up to 17.8% (60–69 years), before declining again in the oldest age groups (7.1% in ≥80 years).

#### 3.2.3. RSV-Related Hospitalizations Requiring Mechanical Ventilation 2017–2023

The need for mechanical ventilation largely paralleled ICU admission trends (Figure 3 and Supplementary Figures S2 and S4). Children aged 0–9 years accounted for 60% of the ventilated cases in the seven-year period (1317/2179), but their ventilation rate was comparatively low (5.6%). Individuals aged 20 to 49 years showed low to moderate ventilation rates (3.0–7.2%), while rates increased in adults aged 50 and older, peaking at 12.6% in the 60–69-year age group, then declining to 9.8% (70–79-years) and 4.5% (≥80 years).

#### 3.2.4. In-Hospital Mortality in RSV-Related Hospitalizations 2017–2023

Overall, 656 RSV-related hospitalizations resulted in in-hospital death (1.8%). While the in-hospital mortality rates remained low in children and younger adults up to 39 years (<2%), they rose progressively with age: between 2.4–2.9% in those aged 40–59 years, exceeding 5% from age 60 onward, and peaking at 7.1% in the oldest age group (≥80 years) (Figure 3 and Supplementary Figure S5).

### 3.3. RSV Infection as Primary vs. Secondary Diagnosis and Additionally Coded Diagnoses

**Figure 4:**
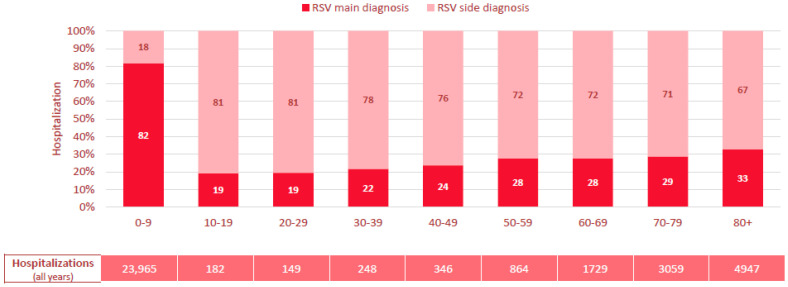
Proportion of RSV infection coded as primary vs. secondary diagnosis in % of overall RSV-related hospitalizations in Switzerland, 2017–2023, stratified by age group in 10-year increments. RSV: respiratory syncytial virus, Hospitaliz.: Hospitalizations.

A clear age-related pattern was also observed in the coding of RSV infection as a primary versus secondary diagnosis (Figure 4). In children aged 0–9 years, RSV infection was recorded as the primary diagnosis in 82% of cases, suggesting it was the main reason for hospitalization, whereas in individuals aged ≥10 years, it was mostly coded as a secondary diagnosis. The proportion of cases with RSV infection coded as the primary diagnosis gradually increased with age, reaching 33% in those aged ≥80 years, while the proportion of RSV infection as a secondary diagnosis declined accordingly (Figure 4).

To better understand the clinical context, we examined the most frequent additional diagnoses coded alongside RSV-related diagnoses, regardless of whether RSV infection was coded as the primary or secondary diagnosis (Supplementary Table S1).

In young children, these were predominantly acute respiratory or infection-related conditions, such as respiratory failure (J96, 44.4%), SARS-CoV-2 testing procedures (U99, 31.1%), and feeding/fluid-related symptoms (R63, 24.5%). Diagnoses such as acute bronchiolitis (J21), acute bronchitis (J20), otitis media (H66, H65), and volume depletion (E86) were also frequently observed. These findings reflect the acute nature of RSV-related hospitalizations in this age group and a tendency towards broader diagnostic coding in pediatric care.

Adolescents showed more heterogeneous patterns. While SARS-CoV-2 testing procedures (U99, 9.3%) and respiratory failure (J96, 7.7%) remained common, several chronic and severe conditions like asthma (J45, 8.2%), epilepsy (G40, 6.6%), lymphoid leukemia (C91, 2.7%), and post-transplant status (Z94, 2.7%) emerged. The frequent occurrence of hematologic malignancies and transplant-associated diagnoses underscores the vulnerability of affected patients, a small but often immunocompromised, patient group.

Among adults, additional diagnoses reflected chronic comorbidities, such as COPD (J44, 17.1%), primary hypertension (I10, 10.8%), heart failure (I50, 10.6%), atrial fibrillation (I48, 8.3%), and chronic kidney disease (N18, 8.2%). Bacterial pneumonia (J15, 6.5%) and electrolyte disorders (E87, 6.2%) were also common. These patterns highlight that RSV-related hospitalizations in older, multimorbid populations often involve decompensation of underlying chronic diseases rather than RSV as the sole cause of admission.

### 3.4. Chronic Comorbidities in Patients with RSV-Related Hospitalizations

To assess clinical vulnerability of patients affected by RSV infection, we analyzed the prevalence of chronic comorbidities and severe conditions among all RSV-related hospitalizations from 2017 to 2023. The analysis included chronic diseases or severe conditions observed at a frequency of ≥0.1% in children, and ≥1% in adolescents and adults.

In children aged 0–9 years, chronic comorbidities were rare (<3% of cases), with the most common being congenital malformations of cardiac septa (Q21, 0.7%), epilepsy (G40, 0.6%), gastroesophageal reflux disease (K21, 0.5%), and asthma (J45, 0.5%). A small subset had more complex conditions such as Down syndrome (0.3%), congenital malformations (of multiple systems 0.3%, of great arteries 0.3%, of head, face, spine and thorax 0.2%) and cerebral palsy (0.2%) (Figure S6, Supplementary Table S2).

Among adolescents (10–19 years), chronic conditions were more prevalent, most commonly asthma (J45, 8.2%) and epilepsy (G40, 6.6%), along with severe or immuno-compromising conditions such as lymphoid leukemia (C91, 2.7%), post-transplant status (Z94, 2.7%), and cerebral palsy (G80, 2.2%). More rare diagnoses such as aplastic anemia (D61, 1.6%), sickle-cell disease (D57, 1.1%), congenital malformations (Q67, 1.1%, Q87, 1.1%), and Down syndrome (Q90, 1.1%) suggest that adolescents hospitalized with RSV infection frequently had serious underlying chronic disease, potentially contributing to longer hospital stays and high ICU admission rates in this age group (Figure S7, Supplementary Table S2).

**Figure 5:**
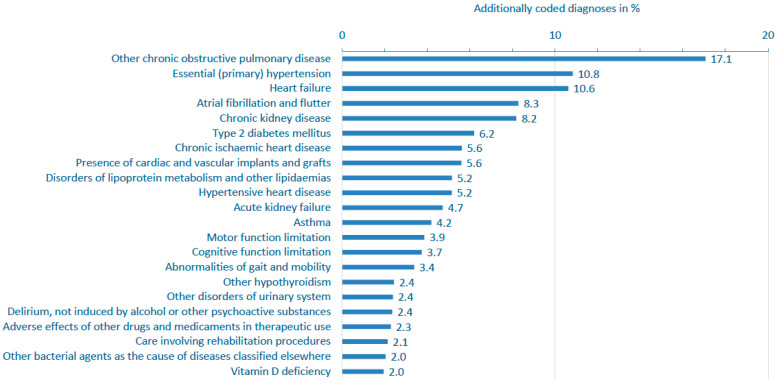
Prevalence of additionally coded diagnoses in RSV-related hospitalizations (primary or secondary diagnoses) in adults aged ≥20 years in Switzerland, 2017–2023. Analysis focused on chronic comorbidities, severe or frailty-associated conditions. All conditions shown had a prevalence of ≥2.0% in the study population. See Supplementary Table S2 for more complete ICD-10 code listings and prevalence across other age groups. RSV: respiratory syncytial virus.

In adults, chronic comorbidities were frequent. Common diagnoses included COPD (J44, 17.1%), hypertension (I10 and I11 combined 16.0%), heart failure (I50, 10.6%), atrial fibrillation or flutter (I48, 8.3%), chronic kidney disease (N18, 8.2%), type 2 diabetes mellitus (E11, 6.2%), and asthma (J45, 4.2%) (Figure 5, Supplementary Table S2).

Beyond common major comorbidities, a wide range of additional conditions was observed, including cardio-metabolic disorders (e.g., chronic respiratory insufficiency, chronic ischemic and hypertensive heart disease, lipid metabolism disorders, hypothyroidism, and kidney dysfunction). Several diagnostic codes reflected frailty and complex care needs, including cognitive and motor impairments, mobility limitations, delirium, vitamin D deficiency, and dehydration or volume depletion. Documentation of prior medical interventions, vascular implants, and the need for rehabilitation further highlighted the patients’ functional vulnerability. Frequent coding of screening and follow-up procedures, particularly for infectious diseases, underscores the clinical complexity of adults hospitalized with RSV infection.

## 4. Discussion

Previous studies have shown that particularly in older adults, RSV infections are typically more severe than influenza, despite occurring less frequently [30,31,32]. Older adults hospitalized with RSV infection tend to experience longer hospital stays, higher rates of ICU admission and mechanical ventilation, and comparable or even higher mortality rates than those hospitalized with influenza [30,31,32].

Our findings demonstrate that RSV-related hospitalizations and outcomes, including LOS, ICU admission, mechanical ventilation, and in-hospital mortality, vary considerably by age. A bimodal distribution emerged: the highest absolute burden, excluding mortality, occurred in children aged 0–9 years and adults aged ≥60 years. While the highest absolute numbers of hospitalizations, ICU admissions, and mechanical ventilation were observed in young children, in-hospital mortality was predominantly observed in older adults, increasing progressively with age. This aligns with previous reports of elevated RSV mortality in elderly populations [33,34]. Although our analysis applied 10-year age increments and therefore did not further stratify within the 0–9-year group, previous research has shown that the majority of RSV-related hospitalizations in children occur in those under 1 year of age, with substantially fewer cases in older children [11,28].

We observed a substantial increase in RSV-related hospitalizations from 2017 to 2023, excluding the COVID-19 pandemic years 2020–2021, when non-pharmaceutical interventions suppressed respiratory virus transmission. During the COVID-19 pandemic, Switzerland implemented a series of strict public health measures, including lockdowns with school and business closures, bans on gatherings, mandatory mask use in indoor settings and on public transport, and the introduction of COVID certificates for access to indoor areas. Starting in spring 2022, these measures were gradually lifted. They were associated with a marked decline in common respiratory infections, which is also reflected in the reduced number of RSV hospitalizations observed in Switzerland during this period [35]. During lockdowns, RSV prevalence among hospitalized children dropped significantly—a meta-analysis reported a drop from 25% to 5% [35]. The inclusion of the years 2020–2021 in our dataset likely lowered the average incidence, leading to an underestimation of the actual RSV burden in Switzerland.

When analyzing outcomes relative to the number of hospitalizations per age group, a nuanced pattern emerged. Although ICU admission rates were highest in the very small overall number of adolescent RSV-related hospitalizations, they were consistently elevated in older adults, particularly those aged 60–79 years. This suggests that, although fewer older adults are hospitalized with RSV infection compared to young children, those who are admitted tend to experience more severe disease, as reflected by higher rates of ICU admission, mechanical ventilation, and in-hospital mortality. The longer length of stay (LOS) observed in adults aged >50 years further underscores the increased clinical complexity and severity in this age group. This observation is likely due to older age and increasing number of comorbidities contributing to complications and more severe outcomes.

In contrast, while hospitalization numbers were highest in children under 10 years, their lower ICU admission, mechanical ventilation and very low mortality rates indicate a generally milder disease course in otherwise healthy pediatric patients.

An unexpected but interesting finding was the high average length of stay and ICU admission rate observed in adolescents, exceeding that of all other age groups. Although this age group accounted for a relatively small number of RSV-related hospitalizations—averaging around 26 cases per year—the severity of outcomes was notable. While previous research suggests that RSV infections in adolescents typically follow a mild clinical course [36], severe presentations can occur in this age group, particularly in individuals with underlying health conditions [37], potentially leading to the indication for intensive care. In our dataset, we observed markedly high numbers of lymphoid malignancies, epilepsy, and post-transplantation status among hospitalized adolescents, possibly explaining the elevated ICU admission rates in this group. Given the low absolute case numbers, small annual fluctuations can lead to large proportional changes, limiting the robustness of age-specific trend interpretation in this population.

In contrast, mortality was almost exclusively observed in patients aged ≥60 years, while those under 40 had very low mortality rates, consistent with the milder disease course in immunocompetent younger adults.

Interestingly, both ICU admission and mechanical ventilation rates declined with increasing age beyond 70, despite rising mortality. This suggests a shift in clinical decision-making, where intensive care is used more selectively in the very elderly—either due to frailty, comorbidity burden, or advance care planning.

A major finding of our study was the high proportion of RSV infection coded as a secondary diagnosis in adults, particularly in those with chronic conditions such as COPD and heart failure frequently listed as the primary diagnosis. This pattern suggests that comorbidities, which are generally more prevalent in older adults, both predispose individuals to a more severe course of RSV infection and may be exacerbated by the infection itself, ultimately contributing to hospitalization.

In contrast to pediatric cases—where RSV infection is typically the sole driver of disease—its role in older adults appears to be more complex, often interacting with underlying respiratory, cardiopulmonary or metabolic conditions and worsening the clinical trajectory [33,38,39,40]. However, our data do not allow us to determine whether RSV infection was the initial trigger for primary respiratory diagnoses such as pneumonia, or whether it was acquired secondarily during hospitalization. RSV infection is a known cause of COPD exacerbations [25,26], and patients with cardiopulmonary conditions have higher rates of RSV-related hospitalizations [24]. Heart failure, frequently associated with pulmonary congestion, may predispose patients to secondary respiratory complications [41,42]. Moreover, chronic heart failure may impair immune function, and consequently increase susceptibility to infections [43]. Recent data from Switzerland also confirmed an association of poor outcome of RSV infection with non-respiratory chronic comorbidities, in particular chronic kidney disease and immunosuppression [44].

Together, these mechanisms likely contribute to the role of RSV infection in the hospitalization of older, multimorbid adults.

Thus, it is plausible that RSV infection plays a central, though under-recognized, role in the clinical deterioration of older adults. This is further supported by the high proportion of secondary RSV diagnoses among all hospitalizations in older age groups, indicating that RSV likely contributed to primary diagnoses such as acute decompensated heart failure and acute COPD exacerbation.

In Switzerland, there is no formal requirement to routinely test patients presenting with respiratory symptoms for RSV infection, which likely contributes to a considerable number of unascertained cases. Even when testing is performed, a relatively high rate of false negatives, depending on the diagnostic method and stage of infection, may lead to additional missed cases [16,17]. These limitations are also reflected in recent European data: one study reported that 57.6% of patients hospitalized with a respiratory tract infection who tested positive for RSV were not assigned an ICD-10 diagnosis for RSV infection during their admission, highlighting the extent of underreporting of RSV-coded hospitalizations [8]. Similarly, a study from France estimated an underreporting of around four- to five-fold of RSV-associated hospitalizations in individuals aged 65 years and older [18]. The magnitude of this under-ascertainment becomes even clearer when comparing the Swiss numbers to recent systematic analyses from other European countries, which estimated RSV-associated hospitalization rates in older adults to be systematically under-estimated in routine data. Johannesen et al. used modelling to demonstrate in five European countries that the number of RSV-associated hospitalizations is significantly higher with increasing age than coded cases suggest (approximately 100 cases per 100,000 inhabitants aged 65–74, 200 in those aged 75–84, and 500 in those aged 85+) [45]. A further analysis by Zhang et al. confirmed these findings showing that, after correction for underreporting, hospitalization rates in individuals 60 years and older are two to six times higher than unadjusted rates [46]. In addition, they reported a higher in-hospital mortality rate of around 10% in this age group [46]. Taken together, these factors strongly suggest that RSV-related hospitalizations are considerably under-ascertained in our study, particularly among older adults.

The increasing prevalence of chronic comorbidities and frailty indicators in our older RSV patient population, together with the rise in RSV infection as a secondary diagnosis, underscores the substantial burden of RSV in this age group. This demonstrates the urgent need for enhanced awareness, improved testing strategies, and preventive approaches—particularly vaccination and risk-adapted clinical management—for older adults.

### Limitations

The study has several limitations related to the use of administrative data. First, reliance on ICD-10 coding may lead to underreporting or misclassification of RSV infection cases, especially given variability in testing and documentation practices across hospitals and over time. Patients with confirmed RSV infection may not have been assigned a specific RSV-related ICD-10 code, and coding as a secondary diagnosis further complicates attribution. The use of diagnosis codes may also under-capture frailty and functional decline, which are often undercoded but clinically relevant in older populations.

Second, causality cannot be inferred from the data. It is unclear whether RSV infection was the primary reason for hospitalization or acquired during the hospital stay, particularly in older adults with multiple comorbidities where RSV infection may exacerbate underlying conditions.

Third, outcomes were limited to in-hospital events. Data on post-discharge mortality, functional recovery, or the need for rehabilitation are lacking, likely underestimating the true burden of RSV.

Furthermore, detailed clinical information on chronic pre-existing conditions (e.g., severity, immunosuppression, frailty) was not available. This is particularly relevant for adolescents aged 10–19-years, where a high proportion of severe cases was observed in a small number of patients in this age group, but could not be fully explained.

Lastly, temporal trends may be influenced by changes in testing behavior, coding practices, or healthcare utilization—especially during the COVID-19 pandemic—limiting interpretability across the full 2017–2023 period.

## 5. Conclusions

In summary, our findings highlight the importance of targeted prevention and management strategies, particularly in pediatric and geriatric populations, as well as in individuals with high risk factors such as cardiopulmonary diseases, where the RSV burden is most pronounced. Switzerland authorized several new RSV prevention options more recently. These include the monoclonal antibody Beyfortus^®^ for newborns and high-risk infants, the protein-based vaccines Arexvy^®^ and Abrysvo^®^, and most recently, the mRNA-based vaccine mRESVIA^®^, all intended for use in adults aged 60 years and older [47,48,49,50]. In addition, Abrysvo^®^ is also approved for maternal immunization between the 32nd and 36th weeks of gestation to confer passive protection to newborns against lower respiratory tract infections caused by RSV [49]. Arexvy^®^ recently received a label extension for use in adults aged 50–59 years who are at increased risk of severe RSV disease [48].

In November 2024, the Swiss Federal Office of Public Health (FOPH) issued recommendation for RSV vaccination in individuals aged ≥75 years and those at high risk of severe disease ≥ 60 years [51]. However, RSV vaccines have not yet been included in the list of reimbursable vaccinations for this group (“Krankenpflege-Leistungsverordnung”) [52] and have not officially been included in the Swiss vaccination schedule [53].

As a result, RSV vaccination must currently be paid out of pocket, which is likely to impair uptake—especially among the elderly population for whom vaccination is recommended. Broader immunization strategies also targeting younger high-risk groups—such as persons with COPD, heart failure, chronic kidney disease, and diabetes mellitus—could further significantly decrease RSV-related hospitalizations, mitigate severe outcomes, and alleviate strain on healthcare resources. A recent Swiss review emphasized the relevance of vaccinations against respiratory infections in patients with chronic lung disease [54].

Future research should aim to identify risk factors for severe RSV progression, refine vaccination strategies, and evaluate the health-economic impact of preventive interventions in adult populations.

## Data Availability

Data is available from the authors upon reasonable request.

## Supplementary Materials

The following supporting information can be downloaded at: https://www.mdpi.com/article/10.3390/v17111407/s1, Figure S1: Annual RSV-related hospitalizations in Switzerland, 2017–2023, stratified by age group. Figure S2: Absolute number of intensive care admissions, mechanical ventilations, and in-hospital mortality in RSV-related hospitalizations in Switzerland, 2017–2023, stratified by age group. Figure S3: Cases of admission to intensive care unit in % of all RSV-hospitalizations, 2017–2023, stratified by age group. Figure S4: Cases of mechanical ventilation in % of all RSV-hospitalizations, 2017–2023, stratified by age group. Figure S5: In-hospital mortality in % of all RSV-hospitalizations, 2017–2023, stratified by age group. Table S1: The 10 most common additionally coded diagnoses (primary and secondary diagnosis) in RSV-related hospitalizations 2017–2023. Figure S6: Prevalence of additionally coded diagnoses in RSV-related hospitalizations (primary or secondary diagnosis) in children aged 0–9 years in Switzerland, 2017–2023. Analysis focused on chronic comorbidities, severe or frailty-associated conditions. Figure S7: Prevalence of additionally coded diagnoses in RSV-related hospitalizations (primary or secondary diagnosis) in adolescents aged 10–19 years in Switzerland, 2017–2023. Analysis focused on chronic comorbidities, severe or frailty-associated conditions. Table S2: Additionally coded diagnoses in RSV-related hospitalizations in Switzerland, 2017–2023, focusing only on chronic, severe or frailty-associated conditions (ICD-10 diagnosis categories) with threshold frequencies at ≥0.1% in young children (0–9 years), ≥1.0% in adolescent (10–19 years) and adults (≥20 years).

## Abbreviations

The following abbreviations are used in this manuscript:

COVID-19	coronavirus disease 2019
FOPH	Federal Office of Public Health
FSO	Swiss Federal Statistical Office
ICU	intensive care unit
LOS	length of hospital stay
RSV	respiratory syncytial virus
WHO	World Health Organization
