# Supplementary material for: Burden and Characteristics of RSV-Associated Hospitalizations in Switzerland: A Nationwide Analysis from 2017 to 2023
Source: Viruses. 2025 Oct 23;17(11):1407. doi: 10.3390/v17111407 (PMC12656979; doi:10.3390/v17111407)
Supplement: Supplementary file 1 [file viruses-17-01407-s001.zip › viruses-3885092-supplementary.pdf]

Burden and Characteristics of RSV-Associated Hospitalizations in Switzerland: A Nationwide Analysis from 2017 to 2023

Supplementary materials

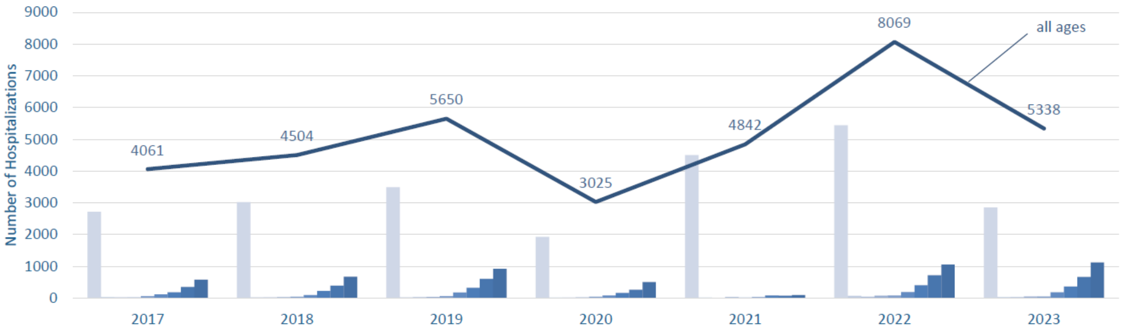

Age group	0–9	10–19	20–29	30–39	40–49	50–59	60–69	70–79	80+	all ages
Hospitalizations (all years)	23,965	182	149	248	346	864	1729	3059	4947	35,489
2017	2720	26	18	19	58	113	181	349	577	4061
2018	3021	19	21	28	43	93	219	386	674	4504
2019	3490	21	25	38	61	174	323	600	918	5650
2020	1930	10	14	22	40	82	159	263	505	3025
2021	4505	12	2	23	16	31	79	76	98	4842
2022	5445	64	39	72	78	192	405	718	1056	8069
2023	2854	30	30	46	50	179	363	667	1119	5338
**average/year**	**3424**	**26**	**21**	**35**	**49**	**123**	**247**	**437**	**707**	**5070**

Figure S1. Annual RSV-Related hospitalizations in Switzerland, 2017–2023. Absolute numbers of hospitalizations per year, stratified by age group. The figure highlights interannual variation, including a marked decline in 2020 during the COVID-19 pandemic, followed by a rebound in 2021 and record-high numbers in 2022–2023. Hospitaliz.: hospitalizations

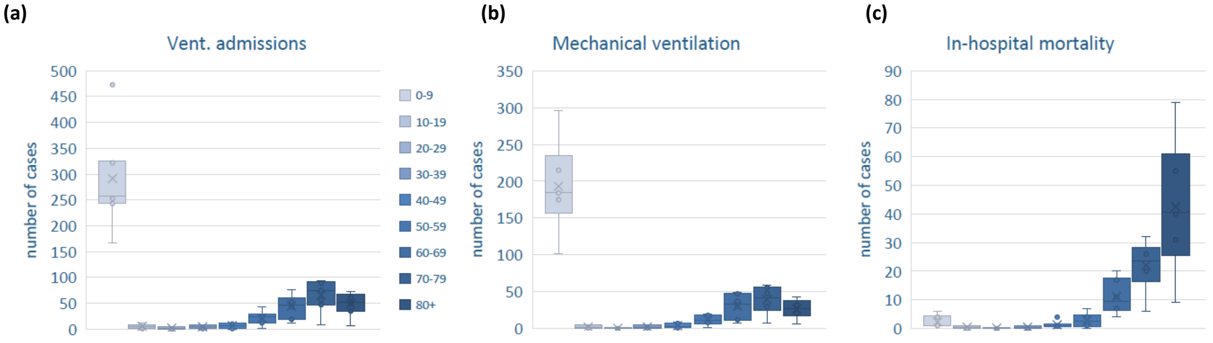

Age group	0–9			10–19			20–29			30–39			40–49		
Hospitalizations (all years)	23,965			182			149			248			346		
Severe Hosp.	Vent.	Vent	Mort	Vent.	Vent	Mort	Vent.	Vent	Mort	Vent.	Vent	Mort	Vent.	Vent	Mort
2017	258	186	1	9	5	1	1	0	0	1	1	0	12	9	1
2018	243	175	1	4	1	1	5	1	0	1	1	1	5	1	1
2019	322	184	4	1	1	0	0	0	0	7	5	1	10	7	4
2020	166	102	1	4	1	0	1	0	0	0	0	0	1	1	0
2021	326	215	1	1	0	0	0	0	0	4	1	1	1	1	1
2022	473	296	6	12	5	1	5	1	1	8	5	0	11	4	1
2023	253	159	5	7	1	0	4	4	1	9	5	1	6	1	1
**average/year**	**292**	**188**	**3**	**5**	**2**	**0**	**2**	**1**	**0**	**4**	**3**	**1**	**7**	**3**	**1**

Age group	50–59			60–69			70–79			80+		
Hospitalizations (all years)	864			1729			3059			4947		
Severe Hosp.	Vent.	Vent	Mort	Vent.	Vent	Mort	Vent.	Vent	Mort	Vent.	Vent	Mort
2017	24	13	1	43	30	8	75	48	27	61	43	31
2018	14	8	1	47	37	7	57	35	20	52	32	41
2019	26	20	7	61	47	20	92	56	32	72	36	55
2020	12	10	4	19	12	11	47	30	21	35	22	40
2021	1	1	0	12	8	4	9	7	6	6	6	9
2022	30	18	4	76	49	17	89	59	26	50	21	79
2023	43	26	7	53	39	17	93	52	37	68	35	87
**average/year**	**21**	**14**	**3**	**44**	**32**	**12**	**66**	**41**	**24**	**49**	**28**	**49**

Figure S2. Absolute number of (a) intensive care admissions, (b) mechanical ventilations, and (c) in-hospital mortality in RSV-related hospitalizations in Switzerland, 2017–2023, stratified by age group. Hospitaliz.: hospitalizations, ICU: intensive care unit, Vent: mechanical ventilation, Mort: in-hospital mortality.

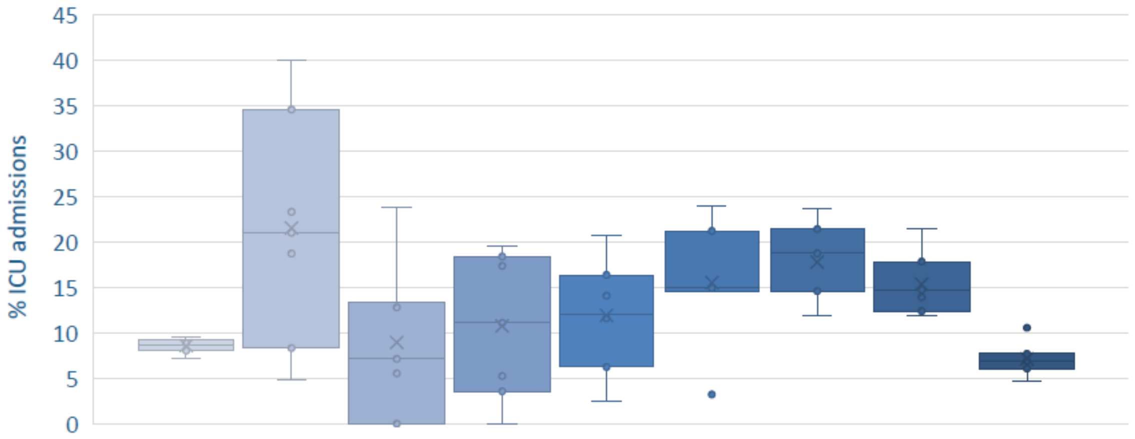

Age group	0–9	10–19	20–29	30–39	40–49	50–59	60–69	70–79	80+
**Hospitalizations** (all years)	23,965	182	149	248	346	864	1729	3059	4947
**2017** (% ICU)	9.5	34.6	5.6	5.3	20.7	21.2	23.8	21.5	10.6
**2018** (% ICU)	8.0	21.1	23.8	3.6	11.6	15.1	21.5	14.8	7.7
**2019** (% ICU)	9.2	4.8	0.0	18.4	16.4	14.9	18.9	15.3	7.8
**2020** (% ICU)	8.6	40.0	7.1	0.0	2.5	14.6	11.9	17.9	6.9
**2021** (% ICU)	7.2	8.3	0.0	17.4	6.3	3.2	15.2	11.8	6.1
**2022** (% ICU)	8.7	18.8	12.8	11.1	14.1	15.6	18.8	12.4	4.7
**2023** (% ICU)	8.9	23.3	13.3	19.6	12.0	24.0	14.6	13.9	6.1
**average/year** (% ICU)	**8.6**	**21.5**	**9.0**	**10.8**	**11.9**	**15.5**	**17.8**	**15.4**	**7.1**

Figure S3. Cases of admission to intensive care unit in % of all RSV-hospitalizations, 2017–2023, stratified by age group. Hospitaliz.: hospitalizations, ICU: intensive care unit.

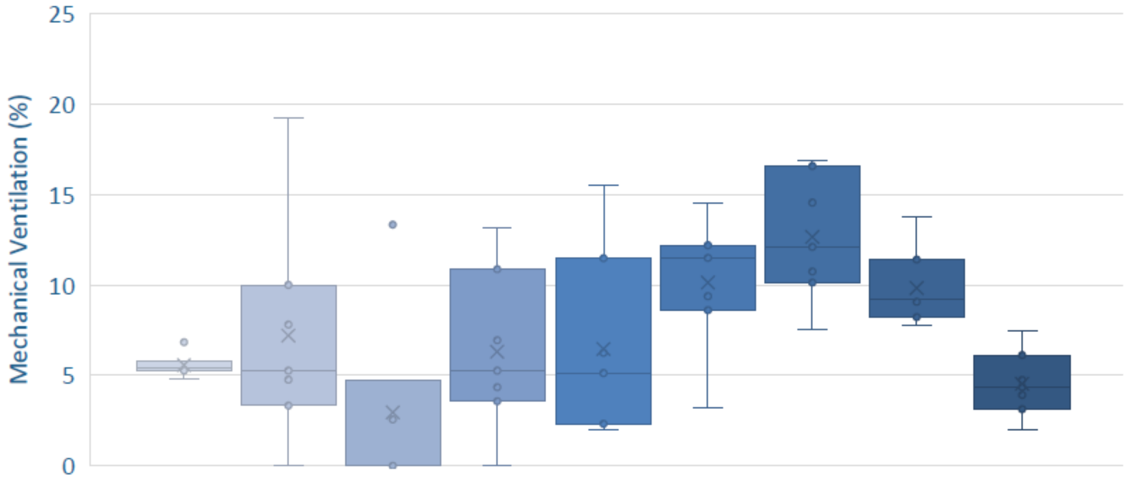

Age group	0–9	10–19	20–29	30–39	40–49	50–59	60–69	70–79	80+
Hospitalizations (all years)	23,965	182	149	248	346	864	1729	3059	4947
**2017** (% Vent.)	6.8	19.2	0.0	5.3	15.5	11.5	16.6	13.8	7.5
**2018** (% Vent.)	5.8	5.3	4.8	3.6	2.3	8.6	16.9	9.1	4.7
**2019** (% Vent.)	5.3	4.8	0.0	13.2	11.5	11.5	14.6	9.3	3.9
**2020** (% Vent.)	5.3	10.0	0.0	0.0	2.5	12.2	7.5	11.4	4.4
**2021** (% Vent.)	4.8	0.0	0.0	4.3	6.3	3.2	10.1	9.2	6.1
**2022** (% Vent.)	5.4	7.8	2.6	6.9	5.1	9.4	12.1	8.2	2.0
**2023** (% Vent.)	5.6	3.3	13.3	10.9	2.0	14.5	10.7	7.8	3.1
**average/year** (% Vent.)	**5.6**	**7.2**	**3.0**	**6.3**	**6.5**	**10.1**	**12.6**	**9.8**	**4.5**

Figure S4. Cases of mechanical ventilation in % of all RSV-hospitalizations, 2017–2023, stratified by age group. Hospitaliz.: hospitalizations, Vent.: mechanical ventilation.

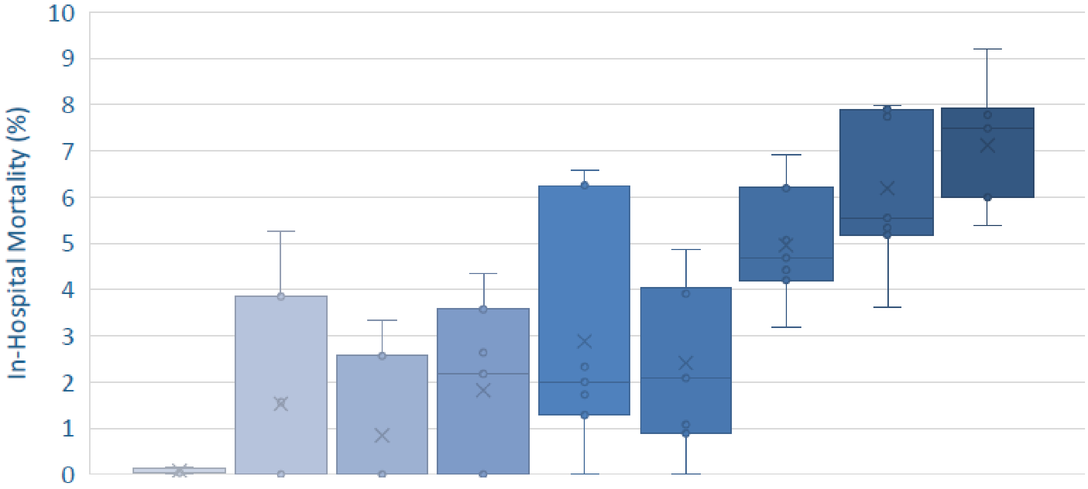

Age group	0–9	10–19	20–29	30–39	40–49	50–59	60–69	70–79	80+
Hospitalizations (all years)	23,965	182	149	248	346	864	1729	3059	4947
2017 (% Mort.)	0.0	3.8	0.0	0.0	1.7	0.9	4.4	7.7	5.4
2018 (% Mort.)	0.0	5.3	0.0	3.6	2.3	1.1	3.2	5.2	6.1
2019 (% Mort.)	0.1	0.0	0.0	2.6	6.6	4.0	6.2	5.3	6.0
2020 (% Mort.)	0.1	0.0	0.0	0.0	0.0	4.9	6.9	8.0	7.9
2021 (% Mort.)	0.0	0.0	0.0	4.3	6.3	0.0	5.1	7.9	9.2
2022 (% Mort.)	0.1	1.6	2.6	0.0	1.3	2.1	4.2	3.6	7.5
2023 (% Mort.)	0.2	0.0	3.3	2.2	2.0	3.9	4.7	5.5	7.8
average/year (% Mort.)	0.1	1.5	0.8	1.8	2.9	2.4	5.0	6.2	7.1

Figure S5. In-hospital mortality in % of all RSV-hospitalizations, 2017–2023, stratified by age group. Hospitaliz.: hospitalizations, Mort.: in-hospital mortality.

Table S1. The 10 most common additionally coded diagnoses (primary and secondary diagnosis) in RSV-related hospitalizations 2017-2023.

ICD-10 category	ICD-10 category description	n (%)
**0-9 years (n = 23,965)**		
J96	Respiratory failure, not elsewhere classified	10,648 (44.4)
U99	Special diagnostic procedures for SARS-CoV-2 testing	7462 (31.1)
R63	Symptoms concerning food and fluid intake	5867 (24.5)
J21	Acute bronchiolitis	2341 (9.8)
Z11	Special screening for infectious and parasitic diseases	1896 (7.9)
H66	Suppurative and unspecified otitis media	1886 (7.9)
H65	Nonsuppurative otitis media	1007 (4.2)
E86	Volume depletion	962 (4.0)
J20	Acute bronchitis	898 (3.7)
J06	Acute upper respiratory infections of multiple or unspecified sites	815 (3.4)
**10-19 years (n = 182)**		
U99	Special diagnostic procedures related to SARS-CoV-2	17 (9.3)
J45	Asthma	15 (8.2)
J96	Respiratory failure, not elsewhere classified	14 (7.7)
G40	Epilepsy	12 (6.6)
J22	Unspecified acute lower respiratory infection	9 (4.9)
E87	Other disorders of fluid, electrolyte and acid–base balance	7 (3.8)
J12	Viral pneumonia, not elsewhere classified	6 (3.3)
A41	Other sepsis	5 (2.7)
C91	Lymphoid leukemia	5 (2.7)
Z94	Organ or tissue replaced by transplant	5 (2.7)
**20+ years (n = 11,342)**		
J44	Other chronic obstructive pulmonary disease	1936 (17.1)
J96	Respiratory failure, not elsewhere classified	1344 (11.8)
I10	Essential (primary) hypertension	1229 (10.8)
I50	Heart failure	1205 (10.6)
I48	Atrial fibrillation and flutter	940 (8.3)
N18	Chronic kidney disease	929 (8.2)
U99	Special diagnostic procedures for SARS-CoV-2 testing	902 (8.0)
Z92	Personal history of medical treatment	863 (7.6)
J15	Bacterial pneumonia, not elsewhere classified	735 (6.5)
E87	Other disorders of fluid, electrolyte and acid–base balance	706 (6.2)

Due to data protection reasons, the accurate number was not available for counts between 1 and 3 cases per age group per reporting year. Thus, the reported numbers in this table represent the lowest possible values, i.e. counting these cases as "1".

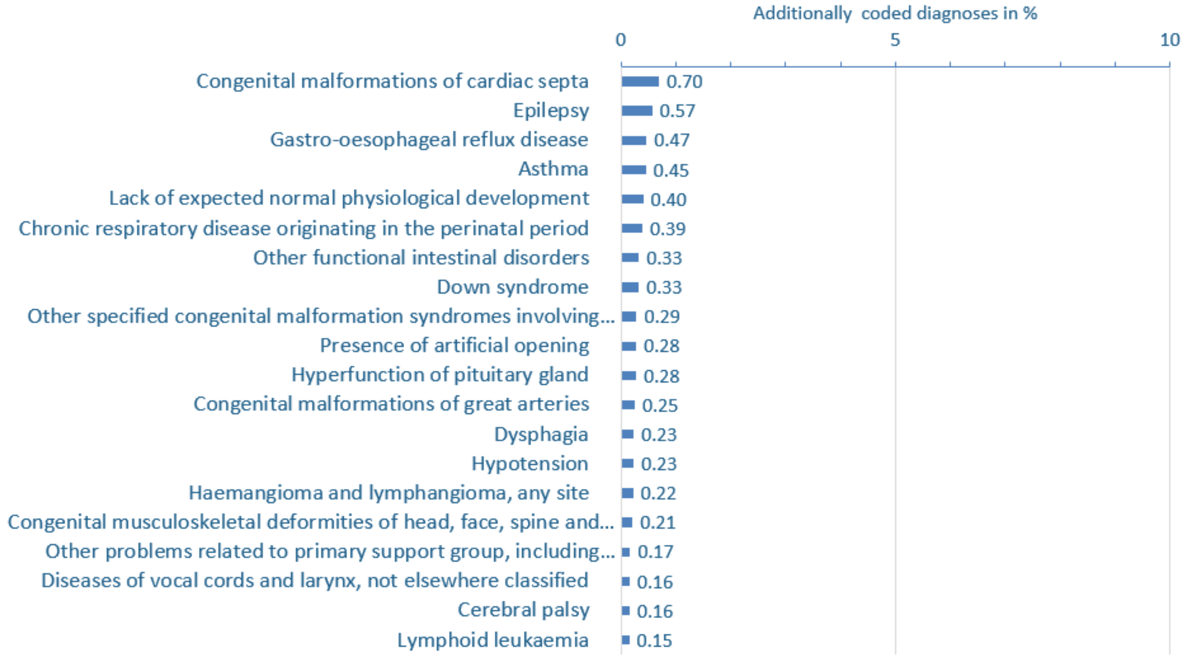

Figure S6. Prevalence of additionally coded diagnoses in RSV-related hospitalizations (primary or secondary diagnosis) in children aged 0-9 years in Switzerland, 2017-2023. Analysis focused on chronic comorbidities, severe or frailty-associated conditions. All conditions shown had a prevalence of ≥0.1% in the study population. See Supplementary Table S2 for more complete ICD-10 code listings and prevalence values across age-groups.

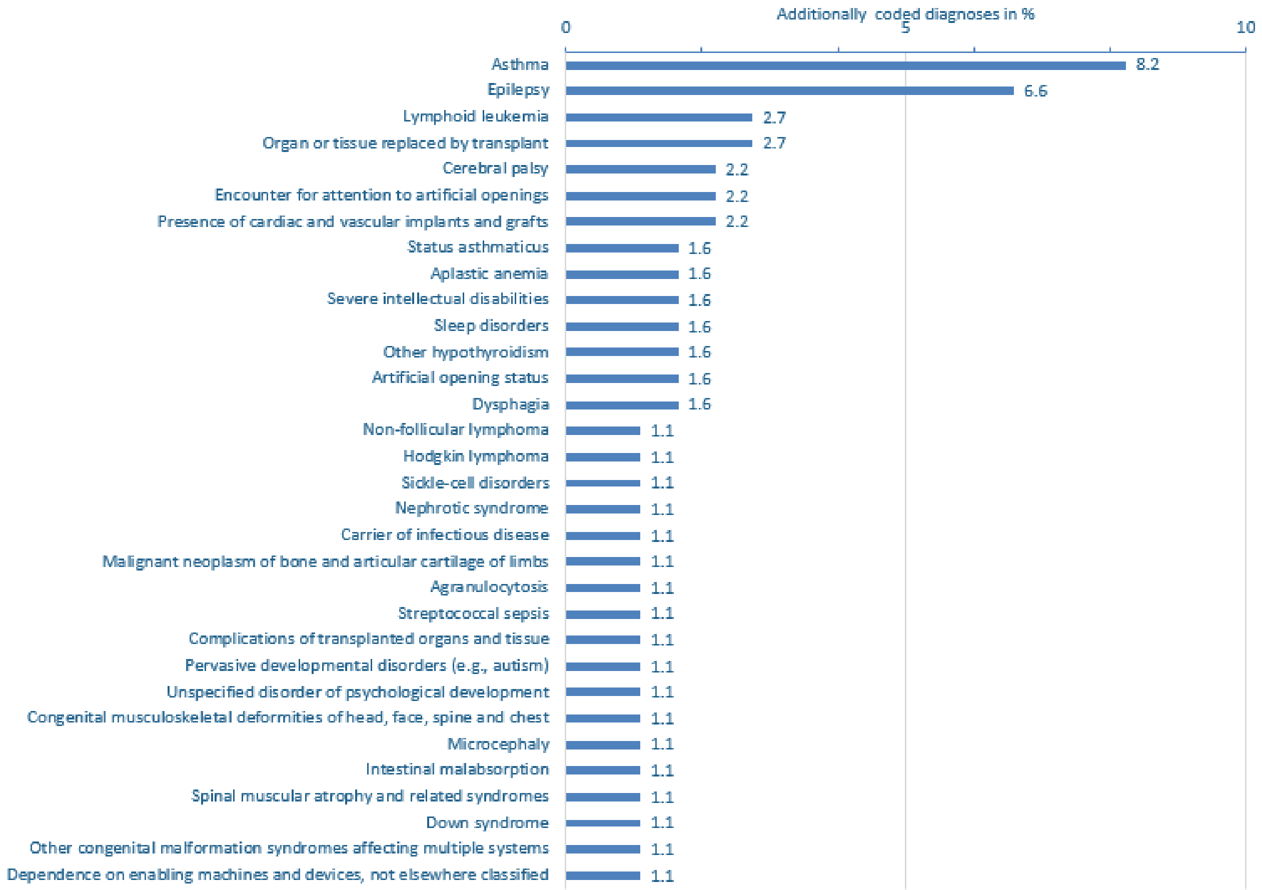

Figure S7. Prevalence of additionally coded diagnoses in RSV-related hospitalizations (primary or secondary diagnosis) in adolescents aged 10-19 years in Switzerland, 2017-2023. Analysis focused on chronic comorbidities, severe or frailty-associated conditions. All conditions shown had a prevalence of ≥1.0% in the study population. See Supplementary Table S2 for more complete ICD-10 code listings and prevalence values across age-groups.

Table S2. Additionally coded diagnoses in RSV-related hospitalizations in Switzerland, 2017-2023, focusing only on chronic, severe or frailty-associated conditions (ICD-10 diagnosis categories) with threshold frequencies at ≥0.1% in young children (0-9 years), ≥1.0% in adolescent (10-19 years) and adults (≥20 years).

ICD-10 category	ICD-10 category description	n (%)
**0-9 years (n = 23,965)**		
Q21	Congenital malformations of cardiac septa	167 (0.70)
G40	Epilepsy	136 (0.57)
K21	Gastro-oesophageal reflux disease	112 (0.47)
J45	Asthma	109 (0.45)
R62	Lack of expected normal physiological development	97 (0.40)
P27	Chronic respiratory disease originating in the perinatal period	94 (0.39)
K59	Other functional intestinal disorders	79 (0.33)
Q90	Down syndrome	78 (0.33)
Q87	Other specified congenital malformation syndromes involving multiple systems	69 (0.29)
Z93	Presence of artificial opening	67 (0.28)
E22	Hyperfunction of pituitary gland	67 (0.28)
Q25	Congenital malformations of great arteries	61 (0.25)
R13	Dysphagia	56 (0.23)
I95	Hypotension	56 (0.23)
D18	Haemangioma and lymphangioma, any site	53 (0.22)
Q67	Congenital musculoskeletal deformities of head, face, spine and thorax	51 (0.21)
Z63	Other problems related to primary support group, including family circumstances	41 (0.17)
J38	Diseases of vocal cords and larynx, not elsewhere classified	38 (0.16)
G80	Cerebral palsy	38 (0.16)
C91	Lymphoid leukaemia	36 (0.15)
Q62	Congenital obstructive defects of renal pelvis and congenital malformations of ureter	34 (0.14)
E03	Other hypothyroidism	33 (0.14)
G47	Sleep disorders	32 (0.13)
P07	Disorders related to short gestation and low birth weight, not elsewhere classified	31 (0.13)
Z43	Encounter for attention to artificial openings	30 (0.13)
L89	Pressure ulcer	30 (0.13)
N39	Other disorders of urinary system	29 (0.12)
Q23	Congenital malformations of aortic and mitral valves	29 (0.12)
Z95	Presence of cardiac and vascular implants and grafts	29 (0.12)
U69	Sequelae or long-term complications related to COVID-19	29 (0.12)
R29	Other symptoms involving nervous and musculoskeletal systems	28 (0.12)
P29	Cardiovascular disorders originating in the perinatal period	28 (0.12)
I27	Other pulmonary heart diseases	26 (0.11)
**10-19 years (n = 182)**		
J45	Asthma	15 (8.2)
G40	Epilepsy	12 (6.6)
C91	Lymphoid leukemia	5 (2.7)
Z94	Organ or tissue replaced by transplant	5 (2.7)
G80	Cerebral palsy	4 (2.2)
Z43	Encounter for attention to artificial openings	4 (2.2)
Z95	Presence of cardiac and vascular implants and grafts	4 (2.2)
G47	Sleep disorders	3 (1.6)
J46	Status asthmaticus	3 (1.6)

D61	Aplastic anemia	3 (1.6)
F72	Severe intellectual disabilities	3 (1.6)
E03	Other hypothyroidism	3 (1.6)
Z93	Artificial opening status	3 (1.6)
R13	Dysphagia	3 (1.6)
C83	Non-follicular lymphoma	2 (1.1)
C81	Hodgkin lymphoma	2 (1.1)
D57	Sickle-cell disorders	2 (1.1)
N04	Nephrotic syndrome	2 (1.1)
C40	Malignant neoplasm of bone and articular cartilage of limbs	2 (1.1)
T86	Complications of transplanted organs and tissue	2 (1.1)
F84	Pervasive developmental disorders (e.g., autism)	2 (1.1)
F89	Unspecified disorder of psychological development	2 (1.1)
Q67	Congenital musculoskeletal deformities of head, face, spine and chest	2 (1.1)
Q02	Microcephaly	2 (1.1)
K90	Intestinal malabsorption	2 (1.1)
G12	Spinal muscular atrophy and related syndromes	2 (1.1)
Q90	Down syndrome	2 (1.1)
Q87	Other congenital malformation syndromes affecting multiple systems	2 (1.1)
Z99	Dependence on enabling machines and devices, not elsewhere classified	2 (1.1)
**20+ years (n = 11,342)**		
J44	Other chronic obstructive pulmonary disease	1936 (17.1)
I10	Essential (primary) hypertension	1229 (10.8)
I50	Heart failure	1205 (10.6)
I48	Atrial fibrillation and flutter	940 (8.3)
N18	Chronic kidney disease	929 (8.2)
E11	Type 2 diabetes mellitus	704 (6.2)
I25	Chronic ischaemic heart disease	638 (5.6)
Z95	Presence of cardiac and vascular implants and grafts	636 (5.6)
E78	Disorders of lipoprotein metabolism and other lipidaemias	586 (5.2)
I11	Hypertensive heart disease	585 (5.2)
J45	Asthma	476 (4.2)
U50	Motor function limitation	439 (3.9)
U51	Cognitive function limitation	425 (3.7)
R26	Abnormalities of gait and mobility	385 (3.4)
E03	Other hypothyroidism	277 (2.4)
N39	Other disorders of urinary system	271 (2.4)
F05	Delirium, not induced by alcohol or other psychoactive substances	268 (2.4)
Y57	Adverse effects of other drugs and medicaments in therapeutic use	260 (2.3)
Z50	Care involving rehabilitation procedures	243 (2.1)
E55	Vitamin D deficiency	222 (2.0)
G47	Sleep disorders	221 (1.9)
M81	Osteoporosis without current pathological fracture	209 (1.8)
D64	Other anaemias	206 (1.8)
I70	Atherosclerosis	196 (1.7)
J98	Other respiratory disorders	194 (1.7)
E44	Protein-energy malnutrition of moderate and mild degree	192 (1.7)
N40	Benign prostatic hyperplasia	172 (1.5)

K21	Gastro-oesophageal reflux disease	168 (1.5)
U69	COVID-19-related delayed complications (national extension)	144 (1.3)
F32	Depressive episode	137 (1.2)
F03	Unspecified dementia	134 (1.2)
J91	Pleural effusion in conditions classified elsewhere	134 (1.2)
R29	Other symptoms and signs involving the nervous and musculoskeletal systems	133 (1.2)
D50	Iron deficiency anaemia	132 (1.2)
I95	Hypotension	132 (1.2)
F41	Other anxiety disorders	128 (1.1)
I35	Nonrheumatic aortic valve disorders	120 (1.1)
J84	Other interstitial pulmonary diseases	116 (1.0)
C90	Multiple myeloma and malignant plasma cell neoplasms	115 (1.0)
G40	Epilepsy	115 (1.0)
D90	Immunodeficiency following chemotherapy	115 (1.0)
N08	Glomerular disorders in diseases classified elsewhere	114 (1.0)
I34	Nonrheumatic mitral valve disorders	113 (1.0)
K59	Other functional intestinal disorders	112 (1.0)
D52	Folate deficiency anaemia	112 (1.0)
I27	Other pulmonary heart diseases	110 (1.0)

Due to data protection reasons, the accurate number was not available for counts between 1 and 3 cases per age group per reporting year. Thus, the reported numbers in this table represent the lowest possible values, i.e. counting these cases as “1”.
